# Investigating Mechanical Properties of Fabricated Carbon-Fiber-Reinforced Composites via LCD Additive Manufacturing

**DOI:** 10.3390/polym15234556

**Published:** 2023-11-28

**Authors:** Anthony Palacio, Mahmoud Baniasadi, Kamran Kardel

**Affiliations:** 1 Manufacturing Engineering Department, Georgia Southern University, Statesboro, GA 30458, USA; ap07917@georgiasouthern.edu; 2 Intel Corporation, Portland, OR 97223, USA; mahmoud.baniasadi@gmail.com

**Keywords:** stereolithography, carbon fiber composites, additive manufacturing, surface functionalization, carbon-fiber-reinforced resin, 3D printing, LCD

## Abstract

Stereolithography (SLA) additive manufacturing is a method of manufacturing capable of generating complex geometric shapes with extremely high accuracy. Classic SLA uses UV curable resins, particularly polylactic acid (PLA), for part generation, but recent research has focused on utilizing this technology for the generation of various composite materials. There has been success in manufacturing composite materials using this technology, but little research has been performed on the generation of carbon-fiber-reinforced composite materials. Carbon fiber stereolithography (CF-SLA) is often overlooked due to carbon fiber’s natural inability to bond with PLA. To overcome this boundary, surface modification techniques were used on chopped carbon fibers to achieve greater bonding. Here, two modification techniques were explored: a sodium dodecyl sulfate (SDS) surfactant addition and nitric acid (HNO_3_) etching. These methods were used to functionalize and prepare the surface of chopped carbon fiber (CF) for bonding with cured PLA resin. Treated fibers were dispersed in generic PLA resin, and tensile test specimens were printed for examining the reinforcement potential of the two treatment methods. Additional complexities arise during printing with fibers including fiber alignment, accumulation, and fiber fallout. To address these issues, a novel in-process mixing method was developed to maintain fiber dispersion. A two-level three-factor factorial design was performed for both treatment methods to determine optimal printing parameters. Through mechanical testing, atomic force microscopy, scanning electron microscopy, and contact angle measurements, the accompanying material property changes were characterized to further develop the field of fiber-reinforced liquid crystal display (LCD) additive manufacturing. After testing, it was found that composites created with SDS nanoparticle modification were stronger than both the acid etched fiber sample and plain PLA. Specifically, SDS surface treatment resulted in a 15% increase in modulus and maximum strength of the sample, mainly by enhancing the interlayer bonding between CF and PLA.

## 1. Introduction

Carbon-fiber-reinforced polymers (CFRP) are a popular field of research as they provide an opportunity to greatly enhance the mechanical properties of various plastics. Carbon fiber (CF) composites exhibit improved mechanical properties, such as high strength and modulus, stiffness, and creep resistance [1]. There is great potential for CFRP to be used in different industries such as replacing steel for bridge cables or underground oil extraction and ocean engineering due to its light weight, high strength, and fatigue resistance. However, there is a need to optimize the process and improve its properties such as durability and cost efficiency to be better adopted by industry [2,3]. This field of research has many implications for additive manufacturing (AM) as demand increases for more complex geometric parts with enhanced mechanical properties. The flexibility AM technologies offer for manufacturing parts with complex geometry and low material waste is unparalleled [4]. One form of AM of particular interest is stereolithography (SLA). Stereolithography additive manufacturing (SLA-AM) was first introduced in 1984 as a system for generating three-dimensional objects layer by layer via UV-curable resin [5]. This method has the capability of generating incredibly accurate and precise parts of complex geometry with relative ease. There are two main styles of SLA, differing only on the direction of printing, and many substyles that differ only on the curing technique. This study focuses on the top-down or inverted SLA printing approach utilizing an LED/LCD curing technique otherwise known as direct UV projecting (DUP). Compared to traditional SLA, DUP printing technology allows for faster build speeds as an entire layer is flashed at once rather than using the complex laser scanning patterns that SLA requires [4,6]. Inherent to most additively manufactured plastics, in comparison to those traditionally manufactured, is a significant reduction in strength and toughness, albeit unilaterally, which can be attributed to the layer-by-layer manufacturing process. Additionally, SLA manufacturing techniques in particular are limited even further by the material properties of available UV curable resins. The unpredictable UV curing process of amorphous crosslinking can result in inconsistent brittleness, limiting the material properties and application of thermosetting polymers [7]. Yet, while SLA products are unpredictable, they maintain status as a viable AM method because of the incredibly high, and consistent, accuracy with which they are produced [8]. It is in this area that the introduction of reinforcement fibers shows significant promise in overcoming this inherent weakness [9,10]. If fiber reinforcement could be used to improve the strength of SLA printed parts, this technology’s applications would be greatly expanded.

The most influential factor in determining the strength of CF-reinforced plastics is the level of bonding between the CF surface and the polymer matrix [11]. Without adequate adhesion, load transfer will not occur when the part undergoes external mechanical stresses. In this situation, fibers can even act as voids or defects in the part resulting in a detriment to mechanical properties. Because of the chemical inertness of untreated or pristine CFs, a weak interfacial bond is created between these fibers and polymer matrices [12,13]. Due to the magnitude of the impact this factor has on the mechanical properties of printed parts, much research has been conducted on potential surface modification techniques that enhance CF–matrix bonding [11,13,14,15,16,17]. This research covers the efficacy of modification techniques such as acid etching, nanoparticle modification, plasma etching, and others. Acid etching is used to change the surface topography of the CF by introducing perforations, pits, and crevasses [18]. This method can also oxidize the surface by introducing various functional groups, which can either assist or inhibit bonding. This has been discussed by Tiwari et al. who reviewed an array of available surface treatment options for carbon fibers [19]. Tiwari et al. also carried out an experiment observing the impact of nitric acid oxidation on the wettability and strength of CFs [20]. Nanoparticle modification modifying the surface of CFs with nanoparticles has been shown to increase the adhesion between CFs and polymer matrices. This method essentially coats the surface of the CF with an element or fiber that can act as a bridge between the CF and the polymer matrix. This bridge increases the level of bonding between the two, which subsequently increases the tensile strength of the final part. Sharma et al. functionalized the surface of CFs with REEs and multi-walled carbon nanotubes (MWNT). This and other research have shown that the texture of the functionalized fiber surface is greatly influenced by nanoparticle modification [18,21]. Additional challenges arise when attempting to print these composites. When dealing with fiber solutions in SLA, dispersion is very important. It is quite common for fibers to mechanically bind and agglomerate in the resin. Additionally, the weight of the fibers often causes them to “fall out” and accumulate at the bottom of the resin tank, resulting in low dispersion throughout the finished part and may also hinder the UV light from curing the resin [10]. Nanoparticle modification can also solve this problem: fibers can be functionalized with a surfactant dispersant to aid in uniform dispersion throughout the resin. While this technique aids in dispersing the fibers, the dispersion is time-dependent and often cannot remain dispersed for the entire print. Pozegic et al. experimented to determine the most effective surfactant dispersant for CF Hi-Per-Dif applications. The results determined that the surfactants sodium dodecyl sulfate (SDS) and sodium dodecylbenzene sulphonate (SDBS) outperformed an array of surfactant dispersants keeping fibers dispersed for the longest time [22]. 

The purpose of this study is to further develop a novel carbon-fiber-reinforced resin for usage in the field of fiber-reinforced stereolithography additive manufacturing. In this study, methods for reinforcing SLA resins through the introduction of modified carbon fibers within the polymer matrix are discussed, tested, and analyzed. The objective is to generate an easy-to-use resin that delivers accurate repeatable results on any SLA machine. This will be accomplished by testing various CF functionalization techniques and creating a design of experiments to determine the optimum parameters for printing these composites.

## 2. Materials and Methods

Only two techniques were chosen for use in this experiment: acid etching and multi-scale nanoparticle modification. The successful techniques shown in the literature were replicated and tested for effectiveness. These fibers were then printed in resin, characterized, and mechanically tested to determine the efficacy of each method for SLA-AM.

### 2.1. Fiber Preparation

The carbon fiber used in this project was sourced from a roll of weaved carbon fiber. Long strands of carbon fiber were taken from a carbon fiber weave, were cut down to size, unsized, and modified in-house using a paper cutter. Based on a design of experiments (DOE), the fibers were cut in two lengths: 6 mm and 12 mm. Because the fibers were sourced from a CF weave, it was assumed that they were sized by the original manufacturer. The sourced fibers were scanned via AFM (Park Systems, NX10, Suwon, Republic of Korea) to verify that they were coated in a sizing agent. Fibers were unsized by placing them in an oven at 500 °C for 3 h [22]. Fibers were then cleaned via ultrasonic bath with acetone for 2 min then rinsed with deionized water. The fibers were scanned again via AFM to verify that the sizing had been effectively removed. After unsizing, these fibers had a diameter of ~8–10 µm. Unsized fibers were also scanned with SEM (JEOL JSM-7600F, JEOL USA, Inc., Peabody, MA, USA) and analyzed with contact angle measurement (Attention Theta Optical Tensiometer, nanoScience Instruments, Phoenix, AZ, USA).

### 2.2. Surface Modification

HNO_3_ etching was chosen based on its success in the literature [20,23]. Following the steps shown by Tiwari and his team, 200 mg of carbon fiber was added to 150 mL of 70% nitric acid and boiled at 110 °C for 60 min [20]. There is a tradeoff in acid fiber etching: as time increases, more etching occurs, yet this process also affects the structural integrity of the fibers. The time of 60 min was proven by Tiwari to be the optimum for etching time, as it increases the interlaminar shear strength with the least amount of fiber breakdown. After fibers were etched, they were thoroughly rinsed in deionized water, then placed in an oven at 100 °C for 5 h to ensure the nitric acid had completely baked off and the fibers were again safe for handling. 

SDS multi-scale nanoparticle modification was also chosen based on results shown in the available literature [22]. Based on their results, SDS was a preferred nanoparticle as it increased both interfacial strength and dispersibility of the CFs. The surfactant dispersant solution of SDS was prepared by dissolving 4.179 g of SDS in 30 mL of deionized water resulting in a concentration of 139.3 mg/mL of SDS. The CFs were cleaned via ultrasound in an acetone solution before applying the nanoparticle. The chopped fibers were weighed out to 30 mg, added to the solution, and tip sonicated for 3 min, 2 s on and 5 s off, at 20% power. Low power ensures no erosion of the CF occurs. The sonicator tip emerged into the solution at 20% as optimally determined by King et al. [24]. Foaming was controlled by adding 1 mL of isopropyl alcohol (IPA) to the solution. The solution was centrifuged for 30 min at 500 rpm, the supernatant was removed, and the remaining fibers were combined with 70% isopropyl alcohol and recentrifuged. The drop-out material was then dried in a vacuum oven at 120 °C for 2 h.

### 2.3. Fabrication via SLA-AM

After functionalization and surface modification, the fibers were added to resin to be printed. The resin chosen was eSun PLA Bio-Photopolymer Clear resin. A bio resin was chosen based on its minimal impact on the environment. The clear resin was chosen to immediately examine carbon fiber dispersion in parts after printing. Fibers were added to 150 mL of resin at two different ratios of 30 and 60 mg. 

All samples were printed on a Uniz Slash 2 LCD/LED 3D printer (UNIZ Technology, San Diego, CA, USA) and post-processed on a Creality3D UW-01 Washing/Curing Station Creality, Shanghai, China). Before printing CF-PLA samples, control samples were printed at two exposure times. All tensile samples were designed according to the ASTM D638 Type IV tensile bar with a chamfer for ease of removal from the build plate [25]. The two levels of exposure time were 4 and 6 s. Once samples were printed, they were removed from the build plate, washed in a clean IPA bath for 2 min, dried at room temperature, and finally post-print UV-cured for 2 min on each side. These samples were covered and stored to eliminate any additional UV exposure that could potentially change the mechanical properties of the printed parts. Parts were stored for 72 h before mechanical testing to ensure full curing.

For each modification technique, functionalized CFs were added to 150 mL of eSun PLA Bio-Photopolymer Clear Resin (Wuhan University, Wuhan, China). Two different solutions with varying weights of CFs were chosen. The information regarding weight percentages and exposure times is shown in Table 1. These samples were post-processed in the same manner as the control samples.

**Figure 1:**
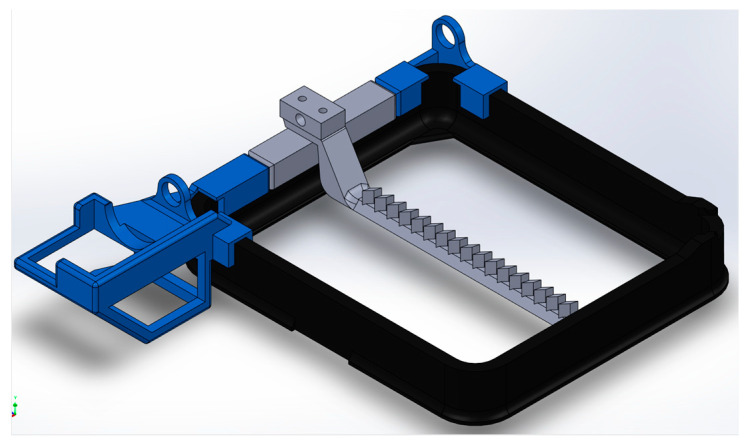
Interlayer continuous kinetic mixing apparatus.

**Figure 2:**
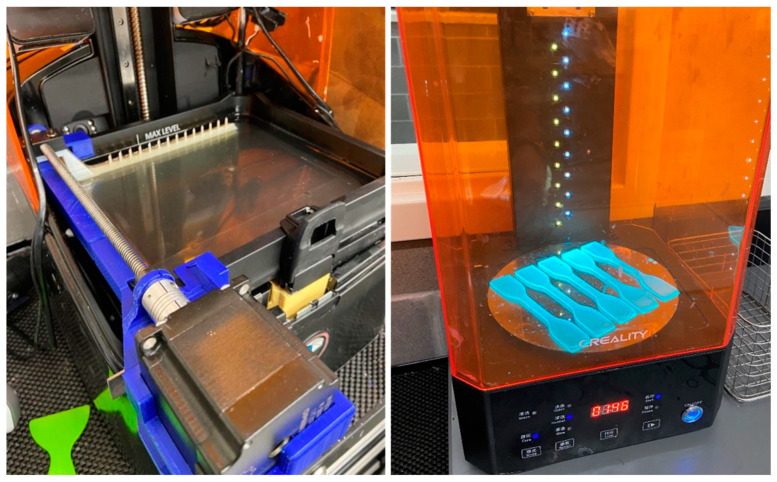
Scraper motor mount final assembly (**left**) and tensile standard parts inside UV post-curing machine (**right**).

To avoid CF settling issues, the CF-PLA mixture was mixed using a custom-designed interlayer continuous kinetic mixing (ICKM) apparatus. ICKM was designed in Solidworks (Dassault Systems, Waltham, MA, USA) to fit the Uniz printer (Figure 1). The structure of ICKM was printed on a Creality CR-10s Pro FDM printer, and the scraper bar was printed on a Stratasys J750 Inkjet printer (Eden Prairie, MN, USA). All circuit components were fabricated in-house and controlled using Arduino’s technology. The scraper spanned the surface of the resin tank and was actuated following each cured layer to disperse and prevent the fallout of the fibers. The final assembly of the ICKM apparatus mounted on the UNIZ SLA printer (UNIZ Technology, San Diego, CA, USA) is shown in Figure 2.

### 2.4. Testing and Characterization

For mechanical testing of the tensile samples, tensile testing was carried out to determine the modulus of elasticity and ultimate tensile strength (UTS) of the parts. Parts were loaded into an MTS Criterion stage and tensile tested with a motor speed of 5 mm/min as specified by ASTM D638 standards [25]. From these tests, the modulus of elasticity and UTS were determined. These results were then compared to each other and the control samples using Minitab statistical software (Version 20.0) to determine the significant factors of the experiment.

Using SEM, modified CFs were examined to observe changes in topography and surface particulate accumulation. Due to CF’s conductive nature, no coating was required for scanning. Treated CFs were spread on a carbon sticker and inserted into the SEM. A high voltage, 30 kV, and low current were used to resolve the highest resolution image. AFM was also used to examine the surface of the CFs before and after surface modifications. A Park NX10 equipped with an AC160TS cantilever was used in non-contact microscopy mode. CFs were spread across double-sided tape to be examined on the AFM. The fiber was examined in non-contact mode to avoid adhesion bias. Visual inspection of each part occurred after printing to examine the dispersion of the CFs in each tensile bar. The fracture surface of select tensile bars was examined to determine whether CFs experienced breakage or pullout from the resin matrix. The fracture surface was coated using gold palladium and examined on a JEOL JSM-6610 SEM. Finally, contact angle measurements were carried out by mounting single fibers on a custom mount and sprayed with fine droplets of deionized water. Contact angle microscopy was carried out in this manner based on the proposed droplet profiling method of Carroll [26] and Yamaki [27]. Using this method, fine droplets are deposited on the surface of the fiber, and the wettability of the different fibers was compared.

## 3. Results

Carbon fiber data and results consisted of raw and treated fiber analysis via SEM, AFM, and contact angle measurement. Tensile bar results consisted of characterization via SEM and tensile testing. 

### 3.1. Atomic Force Microscopy

**Figure 3:**
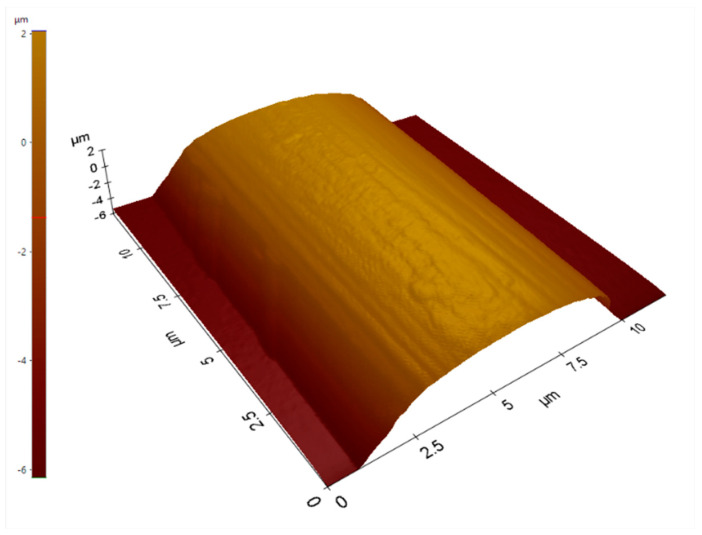
AFM topographic scan of pristine CF.

Pristine unsized fibers were scanned on an AFM to determine the mean diameter and starting surface roughness. An example AFM image of the pristine fiber is shown in Figure 3. The diameter was found to be ~8–10 µm and the roughness was ~0.34 µm. A multi-line measuring tool was used to take averages of the diameter of the fiber. XEI Software version 4.3 (Park Systems, Suwon, Republic of Korea) was used to take both measurements.

**Figure 4:**
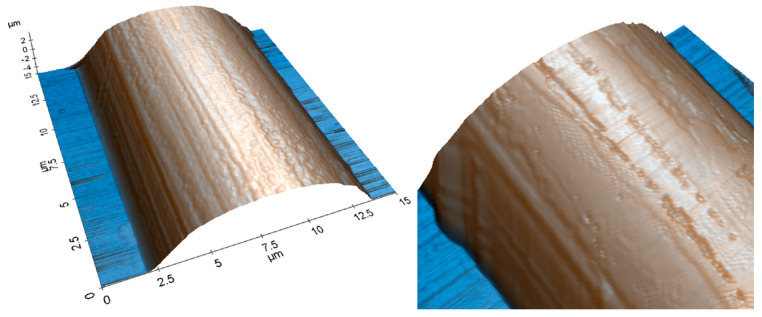
AFM scan of a carbon fiber after surface functionalization with HNO_3_.

The fibers treated with nitric acid etching (HNO_3_) were analyzed under AFM and SEM to show an increased surface roughness over the control samples. AFM scans indicated the fibers had a surface roughness of 0.6 µm. The AFM topography scan is shown below in Figure 4, and the SEM image is shown below in Figure 7. 

**Figure 5:**
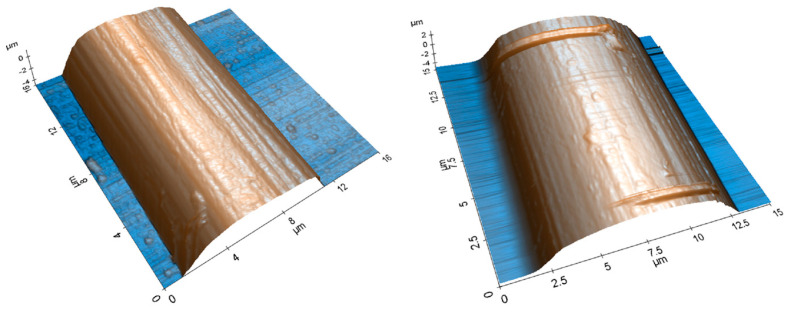
AFM scan of a carbon fiber after surface functionalization with SDS.

Fibers treated via the multiscale nanoparticle method were examined under AFM and SEM to observe the efficacy of the functionalizing process. The AFM scan is shown below in Figure 5, and the SEM scans are shown in Figure 8. AFM scans indicated the fibers had a surface roughness of 0.44 µm. The scans showed many foreign particles attached to the surface of the CFs. These were assumed to be SDS.

### 3.2. Scanning Electron Microscopy

**Figure 6:**
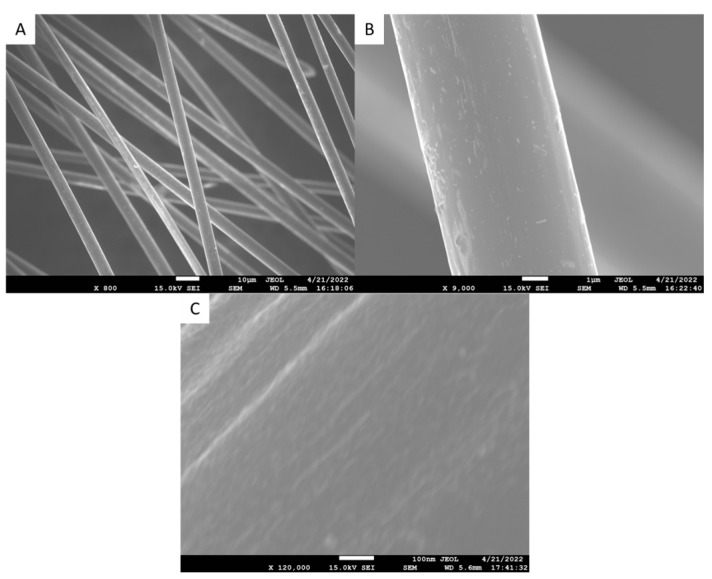
SEM images of raw fibers with 800× (**A**), 9000× (**B**), and 120,000× (**C**) magnification levels.

The same fibers were observed under SEM to visually analyze the surface of the fibers and verify AFM results. SEM images of the raw fibers are shown below in Figure 6.

**Figure 7:**
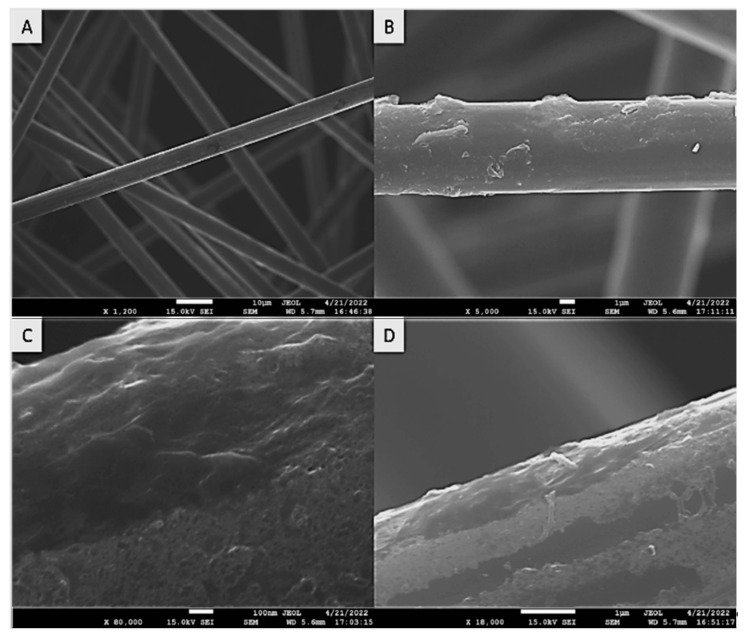
SEM images of carbon fibers after surface functionalization with HNO_3_ with 1200× (**A**), 5000× (**B**), 80,000× (**C**), and 18,000× (**D**) magnification levels.

**Figure 8:**
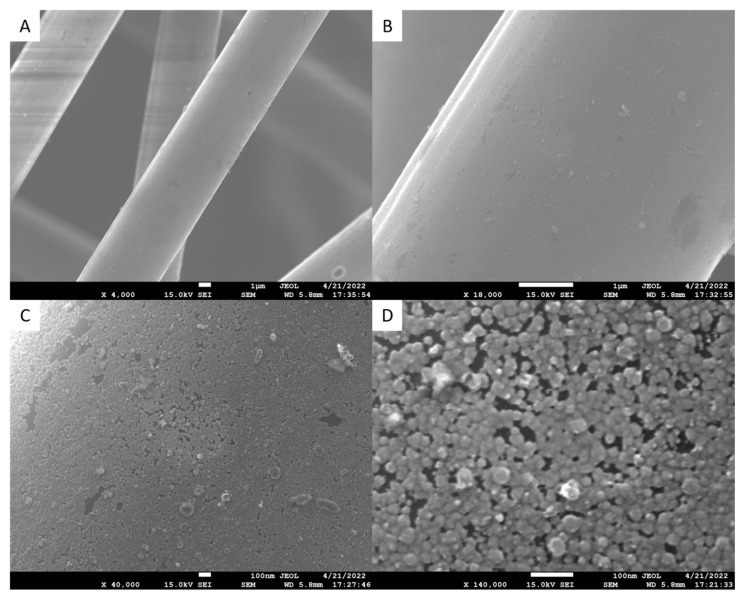
SDS fibers SEM with 4000× (**A**), 18,000× (**B**), 40,000× (**C**), and 140,000× (**D**) magnification levels.

SEM images of fracture surfaces revealed additional information on the interfacial bonding strength of the carbon fibers. A tensile bar from each surface modification group was examined under SEM. 

**Figure 9:**
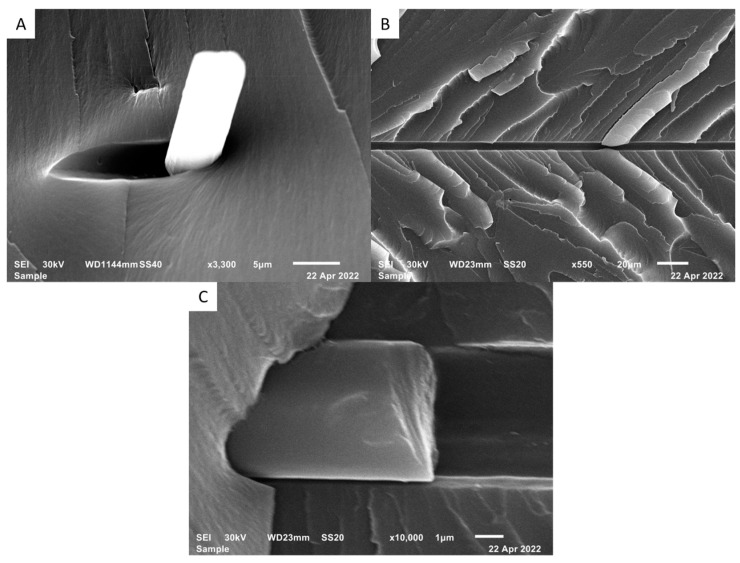
HNO_3_ CF composite fracture surfaces, (**A**) fiber pullout, (**B**,**C**) fiber breaking with two magnification levels.

The fracture surface of the HNO_3_ treated sample is shown in Figure 9. These images suggest two scenarios for fiber failure that were examined on the fracture surface. Fiber pullout was examined in almost every case where the fiber was not aligned in the tensile direction. This resulted in a fracture scenario that was seemingly aided by the fiber acting as a weak link or void in the part. Fiber breakage was observed in the instances where the fibers were aligned less than 45 degrees from the pull direction. A trend was observed of fibers aligning perpendicular to the tensile direction.

**Figure 10:**
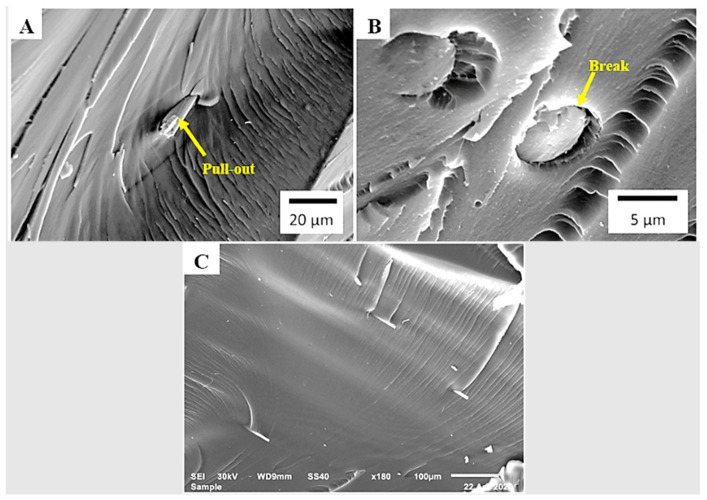
SEM images (**A**) fiber pullout, (**B**,**C**) fiber breaking in different magnification levels (SDS functionalization).

The results of the SDS functionalized tensile sample fracture surface are shown in Figure 10. These images also show the two scenarios for fiber failure on the fracture surface. Similar to the acid-etched samples, fiber pullout was examined in almost every case where the fiber was not aligned in the tensile direction, while fiber breakage was observed in almost every instance that the fibers were aligned in the tensile direction. There was a greater tendency examined for fiber breakage in these samples than that of the acid-etched samples.

### 3.3. Contact Angle Measurement

**Figure 11:**
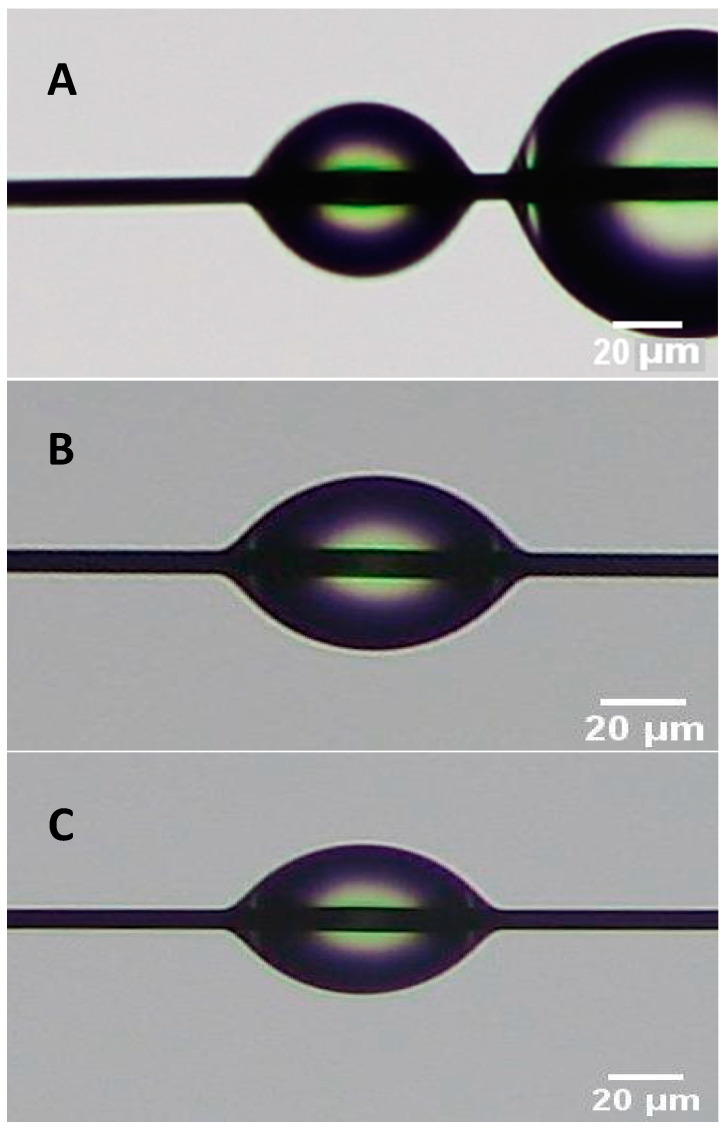
(**A**) Raw carbon fiber contact angle, (**B**) HNO_3_-treated fiber contact angle, and (**C**) SDS-treated fiber contact angle.

Contact angle measurements were taken of the raw carbon fibers. These are shown below in Figure 11A. The measured contact angle for the raw fibers was determined to be approximately 60°.

Contact angle measurements were taken of the HNO_3_- and SDS-treated carbon fibers (Figure 11B,C). The contact angle for the HNO_3_ fibers was approximately 49.5°. The contact angle for the multi-scale nanoparticle surface modification technique (SDS) was approximately 51°.

Due to the complex nature of a single drop on fiber contact angle measurements, the contact angle measurements were compared by only analyzing droplets at a constant height. The contact angle is a function of the height of the droplet, the radius of the fiber, and the length of the droplet. Thus, comparing the contact angle at a constant height and radius results in a relative contact angle. When the height of the droplet was similar to the length of the droplet, the angles were measured to determine if there was a significant change in the contact angle. The results from the contact angle measurement experiment are tabulated below in Table 2.

### 3.4. Tensile Testing

**Figure 12:**
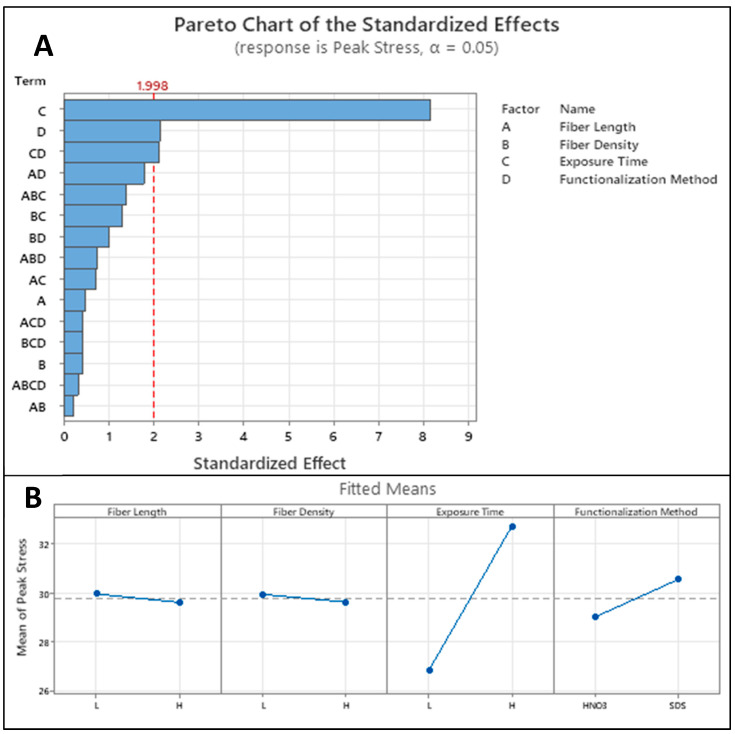
(**A**) Pareto chart and (**B**) main effects plots of peak stress factorial design analysis.

The factorial design was analyzed using Minitab statistical analysis software version 20.0 (Figure 12). From this analysis, the major factors of the experiment were discovered as well as the main effects of these factors. Next, to determine any statistical significance between the highest performing tensile results of the two modification techniques, the results of these tensile tests were compiled into stress–strain curves and analyzed with two-sample t-statistics. The highest performing group from the acid-etched samples was the LHH group, and the highest from the SDS samples was the HLH group. Each modification technique was measured directly against the control samples and against each other. From these graphs, the average toughness of these samples was also calculated by calculating the area under the curve of each graph and taking the average. For the highest performing SDS samples, the average toughness was ~3.81 MPa and ~2.45 MPa for the HNO_3_ samples.

**Figure 13:**
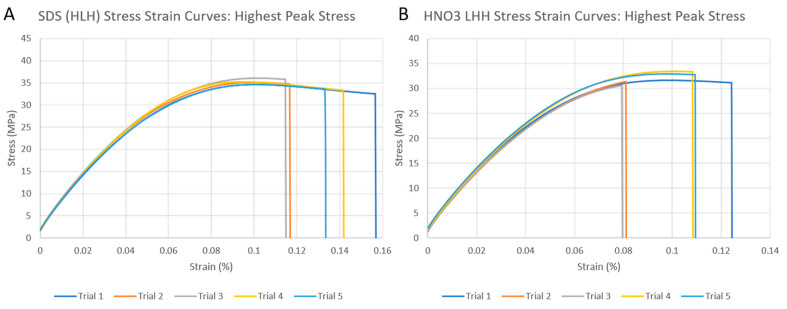
Stress–strain plots for highest performing samples: (**A**) SDS and (**B**) HNO_3_.

Next, to determine any statistical significance between the highest performing tensile results of the two modification techniques, the results of these tensile tests were compiled into stress–strain curves and analyzed with two-sample t-statistics. The highest performing group from the acid-etched samples was the LHH group, and the highest from the SDS samples was the HLH group. Each of the modification techniques was measured directly against the control samples and against each other. An example of the highest performing compiled stress–strain plots for the SDS and HNO_3_ samples are shown in Figure 13. From these graphs, the average toughness of these samples was also calculated by calculating the area under the curve of each graph and taking the average. For the highest performing SDS samples, the average toughness was ~3.81 MPa and ~2.45 MPa for the HNO_3_ samples. Based on Figure 11 and Figure 12, length and density of the fiber did not have direct impact on fracture toughness; however, different functionalization methods (in this case SDS) enhanced fracture toughness. While the authors believe that, in general, for polymers with a pure resin sample would result in more elongation prior to fracture compared to the reinforced CF-resin sample, this could potentially result in higher toughness if the difference in elongation is significant. However, in the case of 3D-printed PLA using LCD 3D printing, the pure resin samples had pretty much similar elongation compared to the CF-PLA samples.

The overall ultimate tensile strength values for the highest-performing groups of both modification techniques and the control samples are shown in Table 3. The values in this table are an average of each set of five samples taken for the different modification techniques and exposure times and their variance.

**Figure 14:**
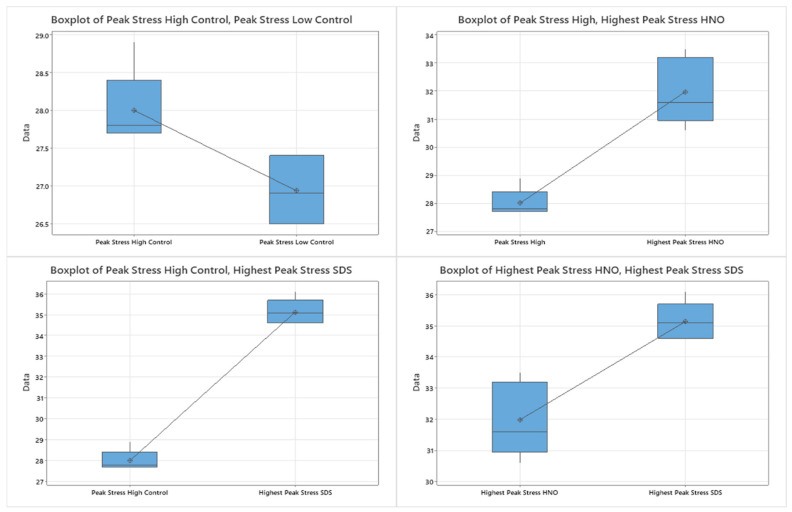
Two sample t-test boxplots.

Minitab was used to generate boxplots to represent the ultimate tensile strength of the tensile samples versus the control samples (Figure 14). Two sample t-statistics with a 95% confidence interval were used to compare the ultimate tensile strengths (UTS) of the CF-reinforced samples and the control samples. For the two exposure times of the control samples, there was a statistically significant difference exhibited, though the UTS differed by a marginal value of ~1 MPa. When comparing both modification groups to the control groups, both groups showed a strong statistical significance. The HNO_3_ samples differed from the control group by ~4 MPa, while the SDS-modified samples had an increased ultimate tensile strength of ~7 MPa. Comparing the two modification groups to one another showed the SDS samples outperformed the acid-etched samples by a statistically significant margin of approximately 3 MPa. 

## 4. Discussion

### 4.1. SLA Printing

Printing of these composites came about with a few issues. Analyzing the continuous kinetic mixer, it was found that while fibers did not accumulate on the bottom of the print bed, after a few layers the fibers would build up on the forward and aft portions of the resin tank. To address this and ensure that there were enough fibers being mixed into the solution for each layer, the fibers were manually mixed into the solution after every 10 layers. The mixing mechanism aligned fibers but not in the hypothesized method. Fibers were aligned in the direction of the scraper bar because of a flow field that was generated in the resin as a result of the resin moving from one side of the tank to the other. As the scraper bar pushed through the tank, due to the high viscosity of the resin, the resin pooled up on one side of the tank. Once the scraper bar reached a certain point, the resin would flow over and through the scraper bar in one quick motion. The flow of the resin in this quick motion generated a flow field that aligned the fibers in the direction of the flow. This was desired for the generation of tensile bars as it opened the possibility for fiber alignment in SLA printing.

### 4.2. Control Group

AFM analysis of raw carbon fibers showed a cohesive surface roughness of 0.33 µm. The surface roughness of this low indicates a very low free surface energy of the raw carbon fibers. Tensile testing showed that as the exposure time of the control samples increased, the ultimate tensile strength of the samples increased. The increase in exposure time, while statistically significant, was not drastic. The high exposure time increased the peak stress by ~1 MPa. Samples printed at exposure times lower than their recommended power density often experience delamination and weaker interlayer bonding. This was true for all samples tested in this experiment. Many samples failed while printing due to delamination, indicating that the lower exposure time did not have enough irradiance at the chosen layer height resulting in weak interlayer bonding. This was represented in all cases of the design of experiment, as seen in Figure 12; the exposure time was the most influential factor.

### 4.3. HNO_3_ Etching

The carbon fibers etched with nitric acid performed better than the control group but did not outperform the SDS nanoparticle modification technique. Fiber analysis of the acid-etched fibers showed a change in the physical topography of the fibers. It was noted that there is a tradeoff in acid fiber etching, as time increases more etching occurs, yet this process also affects the structural integrity of the fibers. The change in the roughness shown by the AFM was of the scale of 0.3 µm. This change in roughness contributed to the increased strength of these composites by increasing the amount of mechanical interlocking that occurs between the fiber and resin matrix. Although the surface of the fiber had higher roughness, the contact angle showed higher wettability. This increase in wettability must then be attributed to generation of functional groups on the fiber surface that increase the surface free energy of the fibers at a greater magnitude than the negative effect of the increase in surface roughness. The etching, then, increased the free surface energy of the fibers via the generation of functional groups on the surface of the fiber and consequently increased the wettability, as shown with the decrease in the relative contact angle of the fibers by approximately 10°. This increase in wettability allows for increased mechanical bonding between the resin and the fibers as the resin is more likely to cover the surface of the fiber. This topographical change is supposed to constitute the increase in ultimate tensile strength of these samples in comparison to the raw PLA control samples. This was verified through the SEM results of the fracture surface. Examination of these fracture surfaces showed almost every fiber found in this set experienced some pullout before breakage. This indicates that the bonding between the resin and CF was lower than desired. The force being applied to the sample at peak load could not be transferred to the fibers, likely because little chemical bonding occurred within the interfacial layer. Thus, the major contributor was a slight increase in friction that was overcome before the peak load was reached. In some cases, this can cause the location of fibers to act like voids in the tensile samples. These “voids” decrease the strength of the composite material in tension. On the contrary, fiber breakage implies that the PLA bonded to the carbon fiber well enough to transfer all energy, up to the tensile strength of the fibers, from the tensile test into the fibers. Although there was a statistically significant difference between these samples and the control samples, the acid-etched group performed worse than the SDS-functionalized group. Acid etching, while very good at changing the topography of the surface, can also affect the mechanical properties of the fibers themselves. Etching can weaken fibers causing them to not retain their original strength. It is likely that this also contributed to these fibers performing worse than the SDS group. 

In this set of tensile samples, as well as the SDS sample set, there was a drastic positive trend in ultimate tensile strength as the exposure time increased. This is contrasted with the only slight increase in strength shown by the control samples. This is likely occurring because the individual layer exposure has the greatest impact on the interfacial layer bonding between the resin and the carbon fibers. As exposure time increases, though the PLA becomes more brittle, the interfacial bonding strength increases, and more energy is transferred to the carbon fibers. This results in a much higher ultimate tensile strength that is unachievable with traditional PLA.

### 4.4. Multiscale Nanoparticle Modification: SDS

SEM scans positively identified a foreign particle on the surface of the SDS-treated fibers as shown in Figure 8. It should be noted that fibers coated with SDS seemed to mix with more ease in the resin matrix and remain dispersed. It was concluded as confirmed in the previous literature that SDS was a preferred nanoparticle as it increased both interfacial strength and dispersibility of the CFs. SEM images of fibers treated with SDS surfactant show fibers covered in a fine layer of particulate spread out across the surface. Again, the increase in wettability can be contributed to the surface free energy added to the surface of the fiber via functional groups, which exceeded the negative impact of increased surface roughness. During SEM analysis of the CF fracture surface, it was observed that in many cases, the CF reinforcement failed by breaking rather than via pullout. Because the primary means of fiber failure was by breaking rather than pullout, it can be concluded that the SDS provided a means of relative effectiveness in bonding the fiber to the resin so that the full strength of the fiber reinforcements may be applied. Though in cases where the fiber was not oriented in the pull direction, the fibers almost always experienced some pullout. Thus, fiber alignment is expected to be a primary factor moving forward in these experiments.

From the results of the tensile experiment, the addition of CF to the resin results in a significant performance increase over the unreinforced sample. The ultimate tensile strength of the samples was seen to increase with CF reinforcement additions. SDS experienced the same positive trend in ultimate tensile strength as that of the nitric-acid-modified samples. From this observation, it can again be hypothesized that increasing the curing time improves the bonding between the fiber and the resin matrix with the help of the surfactant. There was a statistical significance proven with a 95% confidence level that the SDS samples performed better than their nitric acid counterparts. The variance of these samples was much lower than the acid-etched samples resulting in much more consistent results. Because this method achieved the largest increase in mechanical strength of the tensile bars, it is labeled the most effective surface modification technique of the three.

Although the SDS modification technique was tedious to perform, the steps for the generation of the functionalized CF could be easily automated to generate the fibers quickly. This was also the cheapest method of functionalization. Additionally, of the two examined methods, these fibers performed the best in increasing mechanical properties. Because of these characteristics, it is considered for this project the best candidate for use in a commercial carbon-fiber-reinforced resin.

## 5. Conclusions

In this research, two surface modification techniques for carbon fiber were tested and analyzed with the goal of determining an effective method for stereolithography additive manufacturing applications. The two techniques used were nitric acid etching and sodium dodecyl sulfate nanoparticle modification. Characterization of the modified carbon fibers showed that the HNO_3_-etched fibers exhibited physical changes that would aid in bonding, and the SDS-modified fibers showed extensive grafting to the surface of the fibers. Contact angle measurements showed an increase in the surface free energy of these fibers over their raw counterparts. Printing the samples was aided via a continuous kinetic mixing apparatus developed for the purpose of maintaining fiber dispersion throughout the print. This apparatus was successful apart from fiber accumulation on either side of the print bed, which required intermediate mixing. The SDS samples outperformed the HNO_3_ etching technique by a margin of ~3 MPa at its highest tested value. Analyzing the fracture surface of the tensile samples revealed that the fibers oriented perpendicular to the tensile direction pulled out and potentially aided the fracture of the sample, while fibers that were oriented 0–45° from the tensile direction broke and were less likely to slip. It is also hypothesized that fibers oriented 45° from the tensile direction added strength to the sample by adding shear strength to the fibers. The SDS technique showed the greatest potential for increasing the mechanical properties of the PLA samples. This technique is somewhat time consuming as many steps are required for properly modifying the fibers. This technique also resulted in greater mechanical properties when compared to the control group. Because the nanoparticle modification method outperformed the acid-etched fibers and was cheaper to perform, it was chosen as the best candidate analyzed in this study for use in the production of a commercial carbon-fiber-reinforced resin.

## Figures and Tables

**Table 1:** Experimental Parameters.

Modification Technique	Fiber Ratio (g/150 mL)	Fiber Length (mm)	Exposure Time (s)	Samples Printed
Acid Etching: Nitric Acid (HNO_3_)	30	6	4	5
			6	5
		12	4	5
			6	5
	60	6	4	5
			6	5
		12	4	5
			6	5
Multiscale Nanoparticle: Sodium Dodecyl Sulfate (SDS)	30	6	4	5
			6	5
		12	4	5
			6	5
	60	6	4	5
			6	5
		12	4	5
			6	5
None (Control)	N/A	N/A	4	5
			6	5
			Total Samples	90

**Table 2:** Contact angle results (µm).

	Raw	HNO_3_	SDS
Height	50.41	51.19	52.92
Length	65.16	71.40	73.36
Contact Angle	59.07	49.56	50.88

**Table 3:** Highest-performing sample data.

	Control H	HNO_3_ LHH	SDS HLH
Peak Stress Average (MPa)	28.00	31.98	35.14
Variance	0.26	1.417	0.38
Modulus Average (MPa)	1122.39	1315.07	1405.96
Variance	596.88	211.76	470.58

## Data Availability

Data will be made available upon request.
